# Associations between Serum Betaine, Methyl-Metabolizing Genetic Polymorphisms and Risk of Incident Type 2 Diabetes: A Prospective Cohort Study in Community-Dwelling Chinese Adults

**DOI:** 10.3390/nu14020362

**Published:** 2022-01-15

**Authors:** Xiaoting Lu, Rongzhu Huang, Shuyi Li, Aiping Fang, Yuming Chen, Si Chen, Fan Wang, Xinlei Lin, Zhaoyan Liu, Huilian Zhu

**Affiliations:** 1 Department of Nutrition, School of Public Health, Sun Yat-sen University, Guangzhou 510080, China; luxt3@mail2.sysu.edu.cn (X.L.); huangrzh9@mail2.sysu.edu.cn (R.H.); lishy45@mail2.sysu.edu.cn (S.L.); fangaip@mail.sysu.edu.cn (A.F.); chens37@mail2.sysu.edu.cn (S.C.); wangf286@mail2.sysu.edu.cn (F.W.); linxlei3@mail2.sysu.edu.cn (X.L.); 2 Guangdong Provincial Key Laboratory of Food, Nutrition and Health, School of Public Health, Sun Yat-sen University, Guangzhou 510080, China; chenyum@mail.sysu.edu.cn; 3 Department of Medical Statistics & Epidemiology, School of Public Health, Sun Yat-sen University, Guangzhou 510080, China

**Keywords:** betaine, type 2 diabetes, methylenetetrahydrofolate reductase, methyl-metabolizing enzyme, single nucleotide polymorphisms

## Abstract

Previous studies have explored associations between betaine and diabetes, but few have considered the effects of genes on them. We aimed to examine associations between serum betaine, methyl-metabolizing genetic polymorphisms and the risk of type 2 diabetes in Chinese adults. This prospective study comprised 1565 subjects aged 40–75 without type 2 diabetes at baseline. Serum betaine was measured by high-performance liquid chromatography tandem mass spectrometry. Genotyping of methyl-metabolizing genes was detected by Illumina ASA-750K arrays. Cox proportional hazards model was used to estimate hazard ratios (HRs) and 95% confidence intervals (CIs). During a median of 8.9 years of follow-up, 213 participants developed type 2 diabetes. Compared with participants in the lowest quartile of serum betaine, those in the highest quartile had lower risk of type 2 diabetes, adjusted HRs (95%CIs) was 0.46 (0.31, 0.69). For methylenetetrahydrofolate reductase (*MTHFR*) G1793A (rs2274976) and *MTHFR* A1298C (rs1801131), participants carrying 1793GA + AA and 1298AC + CC had lower risk of type 2 diabetes. Interactions of serum betaine and genotype of *MTHFR* G1793A and *MTHFR* A1298C could be found influencing type 2 diabetes risk. Our findings indicate that higher serum betaine, mutations of *MTHFR* G1793A and A1298C, as well as the joint effects of them, are associated with lower risk of type 2 diabetes.

## 1. Introduction

Diabetes is a worldwide health problem with an estimated 463 million adults suffering from this disease in 2019 [1]. It is not only the seventh leading cause of death globally, but also a major cause of heart attacks, stroke, kidney failure, lower limb amputation and blindness [2]. Type 2 diabetes is the most common subtype, accounting for 90–95% of diabetes cases [3]. Previous studies have found that some modifiable factors such as nutrition and lifestyle are associated with the onset of type 2 diabetes [4]. However, investigation of circulating specific nutrients on risk of type 2 diabetes has been limited.

Betaine, also known as trimethylglycine, serves as an important methyl donor in homocysteine methylation in one-carbon metabolism [5]. It has been found that people with diabetes had lower serum betaine levels [6] and disturbed betaine metabolism [7]. However, results from epidemiology studies determined that the associations between betaine and the risk of diabetes are inconsistent [8,9,10,11,12]. One research from the PREVEND (Prevention of Renal and Vascular End-Stage Disease) Study reported that high plasma betaine is associated with low incident type 2 diabetes [11]. Another large prospective cohort study found null associations between dietary betaine intake and the risk of type 2 diabetes [12]. Moreover, few studies have explored associations between serum betaine and incident type 2 diabetes in Chinese population, which has the largest number of people with diabetes in the world, accounting for around 25% of diabetes cases [1].

Circulating betaine may be influenced by several methyl-metabolizing enzymes involved in one-carbon cycle. Catalyzed by choline dehydrogenase (CHDH) or betaine-homocysteine methyltransferase (BHMT), respectively, betaine can be increased by synthesizing from choline pathway, or decreased by transmitting methyl groups to methionine. Owing to the overlap of betaine metabolism and folate metabolism, circulating betaine levels can also be influenced by methylenetetrahydrofolate reductase (MTHFR), which is a key enzyme in folate metabolism [13,14]. Associations between *MTHFR* and both diabetic complications or hereditary diseases have been discussed in previous studies [15,16], but few of them are focused on *MTHFR* and type 2 diabetes. Thus, due to the discrepancies of genes and ethnicity between China and other countries, whether the association between circulating betaine and type 2 diabetes risk in Chinese population is similar with other populations or not remains unclear. Moreover, previous studies have only explored relationships between type 2 diabetes and either betaine or *MTHFR*, investigations of the joint association of them on type 2 diabetes are scant.

Therefore, in this study, our aims were as follows: (1) to examine the relationship between serum betaine levels, relevant methyl-metabolizing enzymes genetic polymorphisms and the risk of incident type 2 diabetes among a large sample size of Chinese adults in Guangzhou Nutrition and Health Study (GNHS); and (2) to explore whether there is a joint association between serum betaine and relevant methyl-metabolizing enzymes genetic polymorphisms with risk of incident type 2 diabetes.

## 2. Material and Methods

### 2.1. Design and Study Population

This study was carried out within the GNHS, an ongoing, community-based prospective cohort study designed to investigate nutrition-related factors associated with risk of chronic diseases, including metabolic diseases and bone health. Detailed protocols of GNHS have been described previously [17]. From 2008 to 2010, 3169 residents aged 40 to 75 years who had been living in Guangzhou for more than five years were successfully recruited via advertisements, posters, health talks and referrals in local communities. Participants returned for follow-up every three years approximately and the third follow-up visit was finished for all participants till 30 April 2019. Study protocol was approved by the Ethics Committee of the School of Public Health of Sun Yat-sen University and was registered at ClinicalTrials.gov (NCT03179657). Written informed consent was provided by each participant before enrollment.

**Figure 1:**
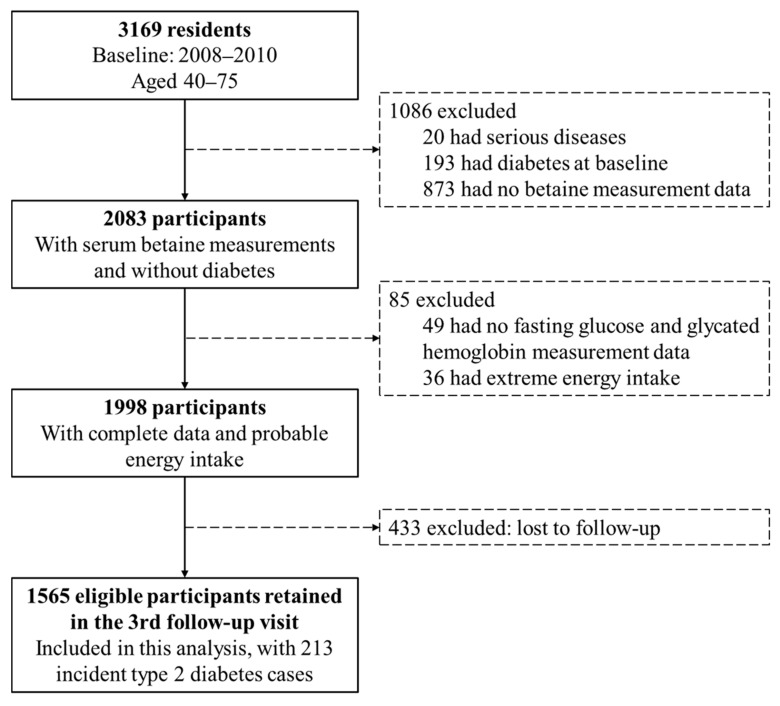
Flowchart of selection of participants from the Guangzhou Nutrition and Health Study.

In this analysis, participants were excluded if they had serious diseases (including cancer, stroke, liver cirrhosis and chronic renal failure. *n* = 20), had diabetes at baseline (*n* = 193), had no serum betaine measurement data (*n* = 873), had no fasting glucose or glycated hemoglobin (HbA_1c_) measurement data (*n* = 49), had extreme energy intake (<500 or >3500 and <800 or >4000 kcal/day for females and males, respectively. *n* = 36) and lost to follow-up (*n* = 433, follow-up rate was 86.3%). The process of participant selection is shown in Figure 1. Finally, 1565 participants were included in the present analysis, of whom 1134 had detected methyl-metabolizing enzymes genetic polymorphisms. The included participants did not differ by most baseline characteristics from those excluded from the analysis (Table S1).

### 2.2. Measurement of Serum Betaine Concentrations

Fasting blood samples were drawn at baseline and were centrifuged, isolated, and stored at −80 °C until analysis. Serum betaine concentrations were measured by high-performance liquid chromatography tandem mass spectrometry (HPLC-MS/MS) (Agilent 6400 Series Triple Quad LCMS, Santa Clara, CA, USA) [18]. Sixty microliters of either the serum samples or standards were added with 100 μL of acetonitrile containing 10 μM internal standards [d9-betaine (Sigma-Aldrich, St. Louis, MO, USA)]. Samples were centrifuged for 10 min at 13,000× *g* to precipitate proteins and then the supernatants were moved into spin columns and centrifuged again for 2 min at 3000× *g* to filter impurities. Finally, the remaining supernatant was transferred to sealed autosampler glass vials, injected into a normal-phase silica column (2.1 mm × 100 mm, 5 μM) by a robotic device and then equilibrated with 30% solution A (15 mmol/L ammonium formate, pH = 3.5) and 70% solution B (acetonitrile). The column was eluted at a flow rate of 0.2 mL/min under isocratic elution. Replicative quality control samples were masked and interspersed among the samples to determine laboratory precision. The coefficient of variation for the between-run assays was 6.21%.

### 2.3. DNA Extraction, Genotyping and SNP Selection

Fasting blood samples were collected with EDTA-coated tubes at baseline and separated into plasma, buffy coat and red cells. DNA was extracted from leukocyte using the TIANamp^®^ Blood DNA Kit according to the manufacturer’s instruction and subsequently stored at −80 °C DNA levels were determined using the Qubit quantification system (Thermo Scientific, Wilmington, DE, USA). Candidate single nucleotide polymorphisms (SNP) we selected were methyl-metabolizing relevant genes, including *MTHFR* G1793A (rs2274976), *MTHFR* A1298C (rs1801131), *MTHFR* C677T (rs1801133), *CHDH* A318C (rs9001) and *BHMT* G742A (rs3733890), which were finally carried out on 1134 subjects with Illumina ASA-750K arrays. Masked quality controls were performed and showed no abnormality. Minor allele frequency (MAF) of these five selected SNPs in this study are all greater than 5% among Chinese population (Table S2).

### 2.4. Covariates Data Collection

A face-to-face structured questionnaire was conducted to collect information on demographical characteristics, lifestyle and diet habits by well-trained personnel at baseline. Education was determined as less than high school, high school and college or above. Household income was grouped into three levels: <1500, 1500–3000, and >3000 yuan/month/person. Smoker was participant who smoked more than 100 cigarettes in his whole life, while alcohol drinker was defined as participant who drank alcohol at least once per week continuously for more than six months of his life. Twenty-four-hour physical activity was assessed using a 19-items questionnaire with the mean metabolic equivalent for activities including occupation, leisure sedentary activity, housework, transportation and physical exercise [19]. Dietary information over the past year was collected using a validated 79-items semi-quantitative food frequency questionnaire [20]. Dietary energy intake (kcal/day) was calculated according to the China Food Composition Database, 2009 [21].

Anthropometric measurements including waist circumference and hip circumference were collected at baseline by experienced research staff using the same calibrated equipment and methods according to standard procedure. Ratio of waist to hip circumference (WHR) was calculated as waist circumference (cm)/hip circumference (cm).

### 2.5. Study Outcomes

Incident type 2 diabetes was the primary endpoint in this analysis and was ascertained up to 30 April 2019. Participants who were with fasting blood glucose concentration ≥ 7.0 mmol/L (126 mg/dL), HbA_1c_ concentration ≥ 6.5% (48 mmol/mol), or self-reported diabetes medications during follow-up visits were considered to have type 2 diabetes, otherwise were considered free of type 2 diabetes. Cutoff values about glucose indexes were ascertained according to the American Diabetes Association criteria for diagnosis [3].

### 2.6. Statistical Analyses

Participants were divided into four groups according to sex-specific quartiles (Q1–Q4) of serum betaine levels, except for analyses stratified by sex. Differences as well as linear trends among baseline demographic, anthropometric and lifestyle characteristics were compared across quartiles of betaine levels by one-way ANOVA or Kruskal-Wallis tests for continuous variables as appropriate and Pearson’s Chi-Squared tests for categorical variables. Hardy-Weinberg equilibrium was calculated to evaluate the genotype distribution by Pearson’s Chi-Squared tests.

Cox proportional hazards models were performed to examine the relationship between both serum betaine levels and selected SNPs, and the risk of type 2 diabetes without adjustment in model 1. Non-modifiable factors, including age (continuous) and sex (females, males), were adjusted in model 2. Further adjustments were conducted for modifiable risk factors of type 2 diabetes, including smoking status (non-smoker, smoker), alcohol drinking (non-alcohol drinker, alcohol drinker), WHR (continuous), physical activity (continuous), energy intake (continuous) in model 3. Hazard ratios (HRs) and 95% confidence intervals (CIs) were calculated with the bottom quartile as the reference. Proportional-hazards assumption was satisfied by including the cross-products of quartiles of serum betaine and time (*p* = 0.245). Linear trends were calculated by assigning quartiles of serum betaine as continuous variables in the models. Non-linearity was performed with restricted cubic splines [22] and no significance was found (*p* = 0.192). Stratified analysis was conducted to examine whether the relationship between quartiles of serum betaine and risk of type 2 diabetes was different in genotypes of various SNPs. Interactions were therefore estimated in multivariate models by including the multiplicative interaction terms. Spearman’s correlation analyses were carried out to explore relationships between serum betaine and SNPs, and all of the correlation coefficients calculated were less than 0.1 (Table S3). Covariates with missing data were imputed by the multiple imputation method.

Statistical analyses were performed using SPSS version 25.0 for windows (IBM Corp., Armonk, NY, USA) or Stata software version 15.0 (StataCorp., College Station, TX, USA). All tests were two-sided and *p* < 0.05 was considered statistically significant.

## 3. Results

### 3.1. Baseline Characteristics of Study Participants

Of the 1565 participants at baseline, there were 1084 (69.3%) females. Mean age was 57.4 ± 4.9 years. Table 1 lists the basic characteristics by sex-specific quartiles of serum betaine levels at baseline. Participants in the highest quartiles of serum betaine had lower WHR compared with participants in the lowest quartile (*p*-trend = 0.014). Education levels of the participants were different among quartiles of serum betaine, but no linear trend was found (*p*-trend = 0.341). Other characteristics including sex, energy intake, physical activity, household income, smoking status, and alcohol drinking did not show significantly difference by quartiles of serum betaine.

### 3.2. Serum Betaine and Risk of Incident Type 2 Diabetes

During 12,168 person years (median follow-up time: 8.9 years) of follow-up, 213 cases (139 of which were females, 65.3%) of type 2 diabetes were identified. Associations between sex-specific quartiles of serum betaine and type 2 diabetes were presented in Table 2. Numbers of incident cases and incidence rates of type 2 diabetes were highest in the bottom quartile and lowest in the fourth quartile of serum betaine. An inverse association between serum betaine levels and type 2 diabetes incidence was found with a HR of 0.45 (95%CI: 0.30–0.66, *p*-trend < 0.001) at the highest quartile compared to the first quartile without adjustment. In multivariable analyses with adjustment for non-modifiable factors, including age and sex, compared with the first quartile of serum betaine, the adjusted HR in the highest quartile was 0.45 (95%CI: 0.30–0.67, *p*-trend < 0.001). The association remained statistically significant after additional adjustment for modifiable risk factors of type 2 diabetes, including smoking status, alcohol drinking, WHR, physical activity and energy intake, and the fully adjusted HR in the highest quartile was 0.46 (95%CI: 0.31–0.69, *p*-trend < 0.001) in comparison with the lowest quartile of serum betaine. Consistent results were found in 1134 subjects with SNPs data (Table S4). Associations of serum betaine levels and type 2 diabetes were similar in females and males (*p*-interaction = 0.806).

### 3.3. Methyl-Metabolizing Enzymes Genetic Polymorphisms and Incident of Type 2 Diabetes

Basic characteristics of five selected SNPs of methyl-metabolizing enzymes are presented in Table S2. The frequencies of the minor alleles were 12.3%, 22.3%, 25.1%, 33.2%, and 35.7% for *MTHFR* G1793A, *MTHFR* A1298C, *MTHFR* C677T, *CHDH* A318C and *BHMT* G742A, respectively. All genotype distributions were in the Hardy-Weinberg equilibrium (*p* > 0.05).

The associations of these SNPs with type 2 diabetes risk are shown in Table 3. Compared with normal type genotype (GG), participants who carry heterozygous or homozygous variants (GA and AA) of *MTHFR* G1793A showed a lower incident risk of type 2 diabetes after full adjustments (HR: 0.54; 95%CI: 0.35, 0.84). Compared with genotypes without mutation (AA) of *MTHFR* A1298C, a significant decreased risk was observed among participants who carry heterozygous or homozygous variants (AC and CC), the fully adjusted HR was 0.61 (95%CI: 0.43, 0.86). The other three SNPs did not show significant associations with type 2 diabetes risk.

### 3.4. Interaction between Serum Betaine Levels and Genetic Polymorphisms

The influence of serum betaine levels for type 2 diabetes across strata of genotype of methyl-metabolizing enzymes genetic polymorphisms is shown in Table 4. Multiplicative interactions were statistically significant between sex-specific quartiles of serum betaine and genotype of *MTHFR* G1793A and *MTHFR* A1298C on associations with type 2 diabetes risk (*p*-interaction < 0.05). When stratified by genotype of *MTHFR* G1793A or *MTHFR* A1298C, stronger associations of serum betaine levels with type 2 diabetes risk were found in those carried heterozygous or homozygous variants. No significant multiplicative interaction was observed between other three SNPs and quartiles of serum betaine.

**Figure 2:**
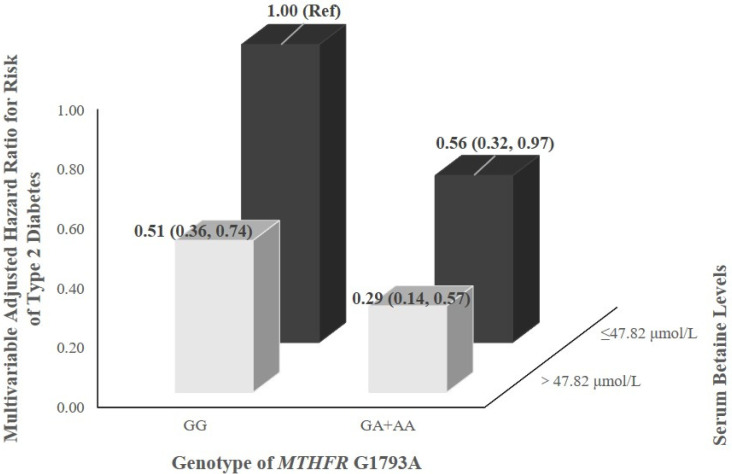

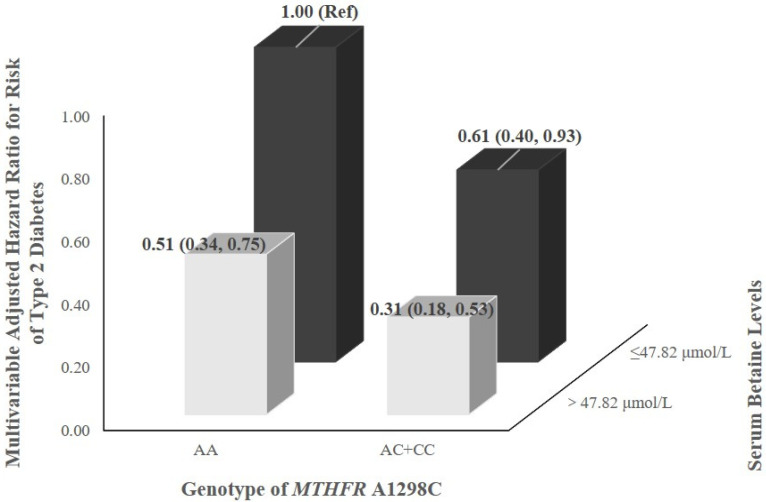
Interaction of serum betaine and genotypes of *MTHFR* G1793A or A1298C on the association with risk of type 2 diabetes in the Guangzhou Nutrition and Health Study.

The joint effects of serum betaine levels and selected SNPs (*MTHFR* G1793A and *MTHFR* A1298C) on risk of type 2 diabetes are shown in Figure 2. After adjusting for non-modifiable and modifiable risk factors of type 2 diabetes, HRs for incident risk of type 2 diabetes were 0.29 (0.14, 0.57) and 0.31 (0.18, 0.53), respectively, for participants with high serum betaine levels (>47.82 μmol/L) and heterozygous or homozygous variants (GA and AA for *MTHFR* G1793A, AC and CC for *MTHFR* A1298C), where the low serum betaine levels (≤47.82 μmol/L) and normal type genotype (GG for *MTHFR* G1793A, AA for *MTHFR* A1298C) was the reference category.

## 4. Discussion

In this prospective cohort study of 1565 community-dwelling Chinese adults, we observed that serum betaine levels were inversely associated with incident of type 2 diabetes after a median follow-up of 8.9 years, while participants who carry heterozygous or homozygous variants of *MTHFR* G1793A and *MTHFR* A1298C had a lower risk of incident type 2 diabetes compared with those carried normal type genotypes. The associations remained robust after adjusting for several potential confounders. Moreover, the joint effects of higher serum betaine levels and heterozygous or homozygous variants of *MTHFR* on reduction of type 2 diabetes risk were also observed.

Some previous studies have investigated the relationship between serum betaine and the risk of type 2 diabetes. In a cross-sectional study of a subset of the NAME (Nutrition, Aging, and Memory in Elders) study, participants with high plasma levels of betaine were associated with lower odds of diabetes after adjusting for covariates [10]. Another study found that increasing betaine levels were associated with lower risk of type 2 diabetes, which indicated that betaine was a marker of type 2 diabetes risk [4]. Similar associations could also be observed in Norwegian, Netherlandish, and Spanish populations [11,23,24]. All of these studies were conducted among American or European population, or participants with certain specific diseases, such as obesity and stable angina pectoris. In the present study carried out among apparently healthy middle-aged and elderly Chinese adults, consistent results were found between serum betaine and risk of type 2 diabetes.

Experimental studies in vitro or in vivo have demonstrated potential biological mechanisms for the relationship between betaine and glucose metabolism. Betaine supplementation not only can prevent the formation or accelerate the consumption of white fat, and therefore inhibit white fat production, but also can reduce intramyocellular lipid accumulation and relieve inflammation, subsequently improving insulin resistance [25]. Additionally, betaine supplementation may increase circulating and hepatic fibroblast growth factor 21 levels, thereby increasing whole-body energy expenditure, maintaining glucose homeostasis and improving insulin resistance [26]. Besides, betaine can inhibit the forkhead box O1 binding to thioredoxin-interacting proteins, which regulates genes involved in cellular metabolic processes and oxidative stress responses, and therefore suppress inflammation and improve insulin resistance [27].

Several genetic variants in human genes have been recognized as risk factors of type 2 diabetes [28]. MTHFR plays an important role in one-carbon cycle and DNA methylation, genetic variations of which may result in disturbances of one-carbon metabolism and DNA methylation. *MTHFR* G1793A and *MTHFR* A1298C are common missense sequence variants of *MTHFR* and diminish MTHFR enzymatic activity [29,30]. Previous studies have explored relationships between *MTHFR* and diseases such as diabetic complications, cancer or hereditary diseases, but few of them are focused on *MTHFR* and type 2 diabetes [15,16,31]. In contrast to our finding, a case-control study including 360 patients and 400 healthy subjects in Tunisia found no significant association between the genotype of *MTHFR* A1298C and type 2 diabetes [32]. Similar associations could be found in a Moroccan population [33]. Results of a meta-analysis, including 6 case-control studies with 852 controls and 897 cases, suggested that 1298CC is a risk factor for type 2 diabetes. Another case-control study including 151 patients and 136 healthy individuals discovered that variants of *MTHFR* A1298C were associated with the risk of type 2 diabetes in Iran’s population [34]. Reasons for these discrepancies could be attributed to differences in genes, ethnicity, study design, sample sizes, as well as covariates using to adjust. Moreover, some previous studies have found that 1793A and 1298C seemed to be a protective factor in several diseases such as male infertility and breast cancer [35,36]. Evidences from a cross-sectional study suggested that variants of *MTHFR* A1298C may protect diabetic patients with low-normal serum folate levels from developing diabetic nephropathy [15]. To some extent, these results implied that *MTHFR* 1793G and 1298A may perform a hazardous function in certain diseases. In addition, we found no association between *CHDH* A318C, *BHMT* G742A and type 2 diabetes risk. Relationships between *CHDH* A318C, *BHMT* G742A, and diseases were less examined in some studies with controversial results. *BHMT* G742A was observed to be associated with decreased risk of colorectal adenoma [37], while no association was found between these two SNPs and breast cancer [38], and the latter was consistent with our results.

Metabolism of betaine, folate, and methionine interweaved on homocysteine in one-carbon cycle. Briefly, homocysteine utilized methyl from betaine or folate cycle to synthesize methionine, and the latter therefore generated S-adenosylmethionine, S-adenosylhomocysteine and finally homocysteine, forming a cycle of methyl metabolism [39]. In our study, we found interactions between serum betaine and genotypes of *MTHFR*. Individuals with high serum betaine levels and heterozygous or homozygous variants had the lowest risk of incident type 2 diabetes. Higher betaine levels could decrease the risk of type 2 diabetes through improving insulin resistance and other biological pathways [25,26,27]. Heterozygous or homozygous state of *MTHFR* may be a protective mutation in several diseases such as diabetes and cancer [15,35]. Betaine served as a main methyl donor in homocysteine transmethylation in the one-carbon metabolism, together with 5-methyltetrahydrofolate (5-methyl-THF), which was determined by MTHFR enzymatic activity. On the one hand, populations with *MTHFR* mutation were frequently accompanied by higher levels of homocysteine [30]. Reduction effects of betaine supplementation on homocysteine could also be observed in *MTHFR* deficient patients, and therefore this improved the prognosis of patients with neurological deterioration [40]. On the other hand, *MTHFR* mutation clearly reduced MTHFR enzymatic activity, and consequently decreased levels of 5-methyl-THF [30]. Previous studies had found that betaine and 5-methyl-THF were regarded as interchangeable sources of methyl groups partly [41]. These suggested an internal relation between betaine and MTHFR enzyme. Levels of circulating betaine and functional pathways it involved may be influenced by enzymatic activities of MTHFR. Further mechanisms need to be certified in future studies.

The strengths of our study are notable. First, we perform a community-based cohort study with large sample size and long follow-up, which is able to investigate the association between serum betaine levels and the risk of incident type 2 diabetes prospectively. Second, concentrations of betaine are measured in serum by using HPLC-MS/MS, which can provide more accurate circulating levels. Third, the frequencies of alleles we selected are semblable to previous studies in the Chinese population [38,42], which confirms less selection bias in our study.

Several limitations should be considered. First, participants are middle-aged and older Chinese individuals, and therefore the generalizability of the findings using to other populations should be applied modestly. Furthermore, despite the fact that we adjusted for multiple potential confounders, including demographic and lifestyle covariate data, residual confounding cannot be ruled out completely. Finally, SNPs we selected in our study are potentially functional with reported MAF > 5% in the Chinese population. Further genome-wide association studies need to be designed to examine relationships between these SNPs and risk of type 2 diabetes.

## 5. Conclusions

In conclusion, higher serum betaine levels are associated with lower risk of type 2 diabetes in middle-aged and older adults in urban areas of southern China. Heterozygous or homozygous genotypes of *MTHFR* G1793A, and A1298C may be protective mutations in developing type 2 diabetes. The joint effects of higher serum betaine levels and heterozygous or homozygous genotypes of *MTHFR* (including *MTHFR* G1793A, and A1298C) on decreasing risk of type 2 diabetes can also be found. Underlying mechanisms of these complicated associations need to be further elucidated in the future.

## Figures and Tables

**Table 1:** Baseline characteristics by sex-specific quartiles of serum betaine levels ^1^.

	Sex-Specific Quartiles of Serum Betaine Levels				*p*	*p*-Trend
	Q1 (*n* = 391)	Q2 (*n* = 393)	Q3 (*n* = 391)	Q4 (*n* = 390)		
Serum betaine, μmol/L						
Females	25.60 ± 7.74	41.58 ± 2.97	50.72 ± 2.91	68.90 ± 13.71	<0.001	<0.001
Males	31.47 ± 8.50	48.35 ± 3.14	58.73 ± 2.98	77.02 ± 12.32		
Age, years	57.59 ± 4.97	57.47 ± 4.92	57.69 ± 5.09	57.26 ± 4.91	0.659	0.504
WHR	0.88 ± 0.07	0.89 ± 0.06	0.88 ± 0.06	0.87 ± 0.07	0.002	0.014
Energy intake, kcal/day	1829.86 ± 534.72	1814.73 ± 480.77	1827.31 ± 507.30	1819.52 ± 489.09	0.973	0.872
Physical activity, MET × hours/day	25.83 ± 6.86	25.50 ± 7.25	25.84 ± 7.85	25.68 ± 6.65	0.901	0.939
Females	271 (69.3)	272 (69.2)	270 (69.1)	271 (69.5)	0.999	0.971
Education levels					0.043	0.341
Less than high school	122 (31.2)	112 (28.5)	124 (31.7)	105 (26.9)		
High school	161 (41.2)	181 (46.1)	183 (46.8)	205 (52.6)		
College or above	108 (27.6)	100 (25.4)	84 (21.5)	80 (20.5)		
Household income, yuan/month/person					0.786	0.624
<1500	147 (37.6)	127 (32.3)	136 (34.8)	139 (35.6)		
1500–3000	176 (45.0)	183 (46.6)	179 (45.8)	175 (44.9)		
≥3000	68 (17.4)	83 (21.1)	76 (19.4)	76 (19.5)		
Smoking status					0.985	0.702
Non-smoker	329 (84.1)	332 (84.5)	331 (84.7)	332 (85.1)		
Smoker	62 (15.9)	61 (15.5)	60 (15.3)	58 (14.9)		
Alcohol drinking					0.247	0.196
Non-alcohol drinker	364 (93.1)	362 (92.1)	373 (95.4)	368 (94.4)		
Alcohol drinker	27 (6.9)	31 (7.9)	18 (4.6)	22 (5.6)		

^1^ Data are mean ± SD or *n* (%). Abbreviations: MET, metabolic equivalent tasks; Q1, first quartile; Q2, second quartile; Q3, third quartile; Q4, fourth quartile; WHR, ratio of waist to hip circumference.

**Table 2:** Hazard ratios (HRs) and 95% confidence intervals (CIs) of type 2 diabetes according to sex-specific quartiles of serum level of betaine (*n* = 1565).

	Sex-Specific Quartiles of Serum Betaine Levels				
	Q1 (*n* = 391)	Q2 (*n* = 393)	Q3 (*n* = 391)	Q4 (*n* = 390)	*p*-Trend
Number of cases	76	58	43	36	-
Person years at risk	2915.77	3063.16	3091.28	3097.66	-
Model 1 ^1^	1.00 (ref)	0.73 (0.52, 1.03)	0.53 (0.37, 0.78)	0.45 (0.30, 0.66)	<0.001
Model 2 ^2^	1.00 (ref)	0.74 (0.52, 1.04)	0.54 (0.37, 0.78)	0.45 (0.30, 0.67)	<0.001
Model 3 ^3^	1.00 (ref)	0.68 (0.48, 0.96)	0.55 (0.38, 0.80)	0.46 (0.31, 0.69)	<0.001

^1^ Unadjusted. ^2^ Adjusted for non-modifiable factors, including age (continuous) and sex (females, males). ^3^ Adjusted additionally for modifiable factors, including smoking status (non-smoker, smoker), alcohol drinking (non-alcohol drinker, alcohol drinker), WHR (continuous), physical activity (continuous), energy intake (continuous). Abbreviations: Q1, first quartile; Q2, second quartile; Q3, third quartile; Q4, fourth quartile.

**Table 3:** Hazard ratios (HRs) and 95% confidence intervals (CIs) of type 2 diabetes according to genotype of five selected genetic variants (*n* = 1134).

	Genotype (*n* = 1134)	
	Non-Mutated Group	Heterozygous or Homozygous Mutated Group
*MTHFR* G1793A (rs2274976)	GG	GA + AA
Cases/*n*.	134/878	24/256
Model 1 ^1^	1.00 (ref)	0.59 (0.38, 0.92)
Model 2 ^2^	1.00 (ref)	0.60 (0.39, 0.92)
Model 3 ^3^	1.00 (ref)	0.54 (0.35, 0.84)
*MTHFR* A1298C (rs1801131)	AA	AC + CC
Cases/*n*.	110/686	48/448
Model 1 ^1^	1.00 (ref)	0.65 (0.46, 0.91)
Model 2 ^2^	1.00 (ref)	0.64 (0.46, 0.90)
Model 3 ^3^	1.00 (ref)	0.61 (0.43, 0.86)
*MTHFR* C677T (rs1801133)	CC	CT + TT
Cases/*n*.	81/645	77/489
Model 1 ^1^	1.00 (ref)	1.31 (0.96, 1.79)
Model 2 ^2^	1.00 (ref)	1.30 (0.95, 1.78)
Model 3 ^3^	1.00 (ref)	1.33 (0.97, 1.81)
*CHDH* A318C (rs9001)	AA	AC + CC
Cases/*n*.	70/503	88/631
Model 1 ^1^	1.00 (ref)	1.01 (0.73, 1.38)
Model 2 ^2^	1.00 (ref)	1.01 (0.74, 1.38)
Model 3 ^3^	1.00 (ref)	0.99 (0.72, 1.36)
*BHMT* G742A (rs3733890)	GG	GA + AA
Cases/*n*.	64/468	94/666
Model 1 ^1^	1.00 (ref)	1.01 (0.74, 1.39)
Model 2 ^2^	1.00 (ref)	1.02 (0.74, 1.40)
Model 3 ^3^	1.00 (ref)	0.91 (0.66, 1.26)

^1^ Unadjusted. ^2^ Adjusted for non-modifiable factors, including age (continuous) and sex (females, males). ^3^ Adjusted additionally for modifiable factors, including smoking status (non-smoker, smoker), alcohol drinking (non-alcohol drinker, alcohol drinker), WHR (continuous), physical activity (continuous), energy intake (continuous). Abbreviations: *BHMT*, betaine-homocysteine methyltransferase; *CHDH*, choline dehydrogenase; *MTHFR*, methylenetetrahydrofolate reductase.

**Table 4:** Adjusted hazard ratios (HRs) and 95% confidence intervals (CIs) of type 2 diabetes according to quartiles of serum betaine levels within each subgroup by related genetic variants (*n* = 1134) ^1^.

		*n*	Quartile of Serum Betaine Levels				*p*-Trend	*p*-Interaction
			Q1 (*n* = 289)	Q2 (*n* = 280)	Q3 (*n* = 286)	Q4 (*n* = 279)		
*MTHFR* G1793A (rs2274976)								0.017
	GG	878	1.00 (ref)	0.71 (0.47, 1.08)	0.47 (0.29, 0.76)	0.40 (0.23, 0.68)	<0.001	
	GA + AA	256	1.00 (ref)	0.34 (0.10, 1.10)	0.35 (0.11, 1.11)	0.33 (0.11, 0.97)	0.039	
*MTHFR* A1298C (rs1801131)								0.007
	AA	686	1.00 (ref)	0.77 (0.48, 1.23)	0.51 (0.30, 0.86)	0.38 (0.21, 0.70)	<0.001	
	AC + CC	448	1.00 (ref)	0.44 (0.21, 0.95)	0.36 (0.16, 0.81)	0.33 (0.15, 0.76)	0.003	
*MTHFR* C677T (rs1801133)								0.086
	CC	645	1.00 (ref)	0.49 (0.28, 0.86)	0.39 (0.21, 0.72)	0.37 (0.20, 0.71)	0.001	
	CT + TT	489	1.00 (ref)	1.01 (0.58, 1.77)	0.57 (0.31, 1.07)	0.38 (0.18, 0.79)	0.003	
*CHDH* A318C (rs9001)								0.787
	AA	503	1.00 (ref)	0.68 (0.38, 1.22)	0.44 (0.22, 0.86)	0.29 (0.14, 0.61)	<0.001	
	AC + CC	631	1.00 (ref)	0.67 (0.39, 1.14)	0.51 (0.28, 0.91)	0.49 (0.26, 0.91)	0.009	
*BHMT* G742A (rs3733890)								0.355
	GG	468	1.00 (ref)	0.74 (0.39, 1.38)	0.56 (0.28, 1.11)	0.37 (0.17, 0.80)	0.007	
	GA + AA	666	1.00 (ref)	0.62 (0.37, 1.02)	0.40 (0.22, 0.71)	0.37 (0.20, 0.69)	<0.001	

^1^ Adjusted for non-modifiable factors, including age (continuous) and sex (females, males), and for modifiable factors, including smoking status (non-smoker, smoker), alcohol drinking (non-alcohol drinker, alcohol drinker), WHR (continuous), physical activity (continuous), energy intake (continuous). Abbreviations: *BHMT*, betaine-homocysteine methyltransferase; *CHDH*, choline dehydrogenase; *MTHFR*, methylenetetrahydrofolate reductase; Q1, first quartile; Q2, second quartile; Q3, third quartile; Q4, fourth quartile.

## Data Availability

Data described in the article are available from the corresponding author upon reasonable request.

## Supplementary Materials

The following supporting information can be downloaded at: https://www.mdpi.com/article/10.3390/nu14020362/s1. Table S1. Baseline characteristics by included and excluded participants; Table S2. Characteristics of the studied SNPs (*n* = 1134); Table S3 Spearman’s correlation coefficients between serum betaine and the studied SNPs (*n* = 1134); Table S4 Hazard ratios (HRs) and 95% confidence intervals (CIs) of type 2 diabetes according to sex-specific quartiles of serum level of betaine in subjects with SNPs data (*n* = 1134).
