# Supplementary material for: Associations between Serum Betaine, Methyl-Metabolizing Genetic Polymorphisms and Risk of Incident Type 2 Diabetes: A Prospective Cohort Study in Community-Dwelling Chinese Adults
Source: Nutrients. 2022 Jan 15;14(2):362. doi: 10.3390/nu14020362 (PMC8778868; doi:10.3390/nu14020362)
Supplement: Supplementary file 1 [file nutrients-14-00362-s001.zip › nutrients-1520200-supplementary.pdf]

Supplementary materials

Associations between Serum Betaine, Methyl-Metabolizing Genetic Polymorphisms and Risk of Incident Type 2 Diabetes: A Prospective Cohort Study in Community-Dwelling Chinese Adults

Table S1. Baseline characteristics by included and excluded participants[1]

	**Included participants** (*n* = 1565)	**Excluded participants** (*n* = 1604)	*p*
Serum betaine, umol/L			
Females	46.69 ± 17.65	45.44 ± 16.89	0.384
Males	53.84 ± 18.22	53.79 ± 19.80	
Age, years	57.51 ± 4.97	57.55 ± 5.36	0.678
WHR	0.88 ± 0.06	0.88 ± 0.07	0.175
Energy intake, kcal/day	1822.85 ± 502.93	1887.83 ± 695.72	0.312
Physical activity, MET×hours/day	25.71 ± 7.16	25.68 ± 7.43	0.765
Females	1084 (69.3)	1180 (73.9)	0.004
Education levels			0.597
Less than high school	463 (29.6)	495 (31.3)	
High school	730 (46.6)	722 (45.6)	
College or above	372 (23.8)	367 (23.2)	
Household income, yuan/month/person			0.786
<1500	549 (35.1)	559 (35.7)	
1500-3000	713 (45.6)	717 (45.8)	
≥3000	303 (19.4)	288 (18.4)	
Smoking status			0.315
Non-smoker	1324 (84.6)	1358 (85.9)	
Smoker	241 (15.4)	223 (14.1)	
Alcohol drinking			0.407
Non-alcohol drinker	1467 (93.7)	1496 (94.4)	
Alcohol drinker	98 (6.3)	88 (5.6)	

[1]Data are mean ± SD or *n* (%). Abbreviations: MET: metabolic equivalent tasks; Q1: first quartile; Q2: second quartile; Q3: third quartile; Q4: fourth quartile; WHR: ratio of waist to hip circumference.

Table S2 Characteristics of the studied SNPs (*n* = 1134)

SNP	Position	Gene	Allel	Genotype [*n* (%)]				MAF(%)	H-W[1]
			M/m	MM	Mm	mm	Mm+mm		
rs2274976	1:11790870	*MTHFR*	G1793A	878 (77.4)	234 (20.6)	22 (1.9)	256 (22.6)	12.3	0.649
rs1801131	1:11794419	*MTHFR*	A1298C	686 (60.5)	390 (34.4)	58 (5.1)	448 (39.5)	22.3	0.973
rs1801133	1:11796321	*MTHFR*	C677T	645 (56.9)	408 (36.0)	81 (7.1)	489 (43.1)	25.1	0.597
rs9001	3:53823890	*CHDH*	A318C	503 (44.4)	508 (44.8)	123 (10.8)	631 (55.6)	33.2	0.977
rs3733890	5:79126136	*BHMT*	G742A	468 (41.3)	523 (46.1)	143 (12.6)	666 (58.7)	35.7	0.995

[1]Calculated with Pearson's Chi-square test. Abbreviations: *BHMT*: betaine-homocysteine methyltransferase; *CHDH*: choline dehydrogenase; H-W: Hardy-Weinberg equilibrium; MAF, minor allele frequency; M/m, major and minor alleles; *MTHFR*: methylenetetrahydrofolate reductase; SNP, single nucleotide polymorphism.

Table S3 Spearman's correlation coefficients between serum betaine and the studied SNPs (n=1134)

r	***MTHFR* G1793A (rs2274976)**	***MTHFR* A1298C (rs1801131)**	***MTHFR* C677T (rs1801133)**	***CHDH* A318C (rs9001)**	***BHMT* G742A (rs3733890)**
Betaine, μmol/L	0.049	0.007	0.023	-0.033	-0.093
Sex-specific quartiles of serum betaine (Q1-Q4)	0.045	-0.002	0.021	-0.041	-0.083

Abbreviations: SNP, single nucleotide polymorphism; *MTHFR*: methylenetetrahydrofolate reductase; *CHDH*: choline dehydrogenase; *BHMT*: betaine-homocysteine methyltransferase; Q1: first quartile; Q2: second quartile; Q3: third quartile; Q4: fourth quartile.

Table S4 Hazard ratios (HRs) and 95% confidence intervals (CIs) of type 2 diabetes according to sex-specific quartiles of serum level of betaine in subjects with SNPs data (*n*=1134)

	**Sex-specific quartiles of serum betaine concentrations**				
	**Q1(*n* = 283)**	**Q2(*n* = 283)**	**Q3(*n* = 285)**	**Q4(*n* = 283)**	***p*-trend**
Number of cases	60	42	31	25	-
Person years at risk	2157.72	2222.67	2342.11	2294.04	-
Model 1[1]	1.00 (ref)	0.68 (0.46, 1.01)	0.48 (0.31, 0.73)	0.39 (0.25, 0.62)	< 0.001
Model 2[2]	1.00 (ref)	0.68 (0.46, 1.01)	0.47 (0.31, 0.73)	0.39 (0.25, 0.62)	< 0.001
Model 3[3]	1.00 (ref)	0.62 (0.41, 0.92)	0.48 (0.31, 0.74)	0.39 (0.24, 0.62)	< 0.001

[1]Unadjusted. [2]Adjusted for non-modifiable factors, including age (continuous) and sex (females, males). [3]Adjusted additionally for modifiable factors, including smoking status (non-smoker, smoker), alcohol drinking (non-alcohol drinker, alcohol drinker), WHR (continuous), physical activity (continuous), energy intake (continuous). Abbreviations: Q1: first quartile; Q2: second quartile; Q3: third quartile; Q4: fourth quartile.
